# Implicit benefits of adolescents with high psychological resilience in action control of emotion regulation

**DOI:** 10.1371/journal.pone.0332384

**Published:** 2025-09-16

**Authors:** Zengyan Yu, Xinghua Lai, Yuqi Wang, Yao Wang, Huitong Zhao

**Affiliations:** 1 School of Mental Health, Qiqihar Medical College, Qiqihar, China; 2 Key Laboratory of Precise Diagnosis and Neuropsychological Regulation of Mental Disorders, Qiqihar, China; Liaoning Normal University, CHINA

## Abstract

Psychological resilience is crucial for adolescents’ emotional health. The aim of this study was to evaluate the relationship between psychological resilience and implicit emotion regulation. The action control theory was used as a model reference. Experiment 1 employed an emotion regulation-implicit associations task using a sample of 56 adolescents and was designed to compare implicit attitudes toward emotion regulation between individuals with high and those with low psychological resilience. The results reveal that adolescents with high psychological resilience are more inclined to use controlled emotion regulation to regulate their emotions. Experiment 2 was an indirect examination of the differences between the implicit emotion repair effects of adolescents with high and those with low psychological resilience (n = 75). The findings indicate that adolescents with high (vs. low) psychological resilience search faster to detect happy faces in an angry context. The results of the two experiments consistently suggest that adolescents with high psychological resilience have an implicit advantage in emotion regulation, which contributes to their emotional health.

## Introduction

In recent years, against a backdrop of rapid societal changes and intensifying academic competition, mental health issues have become increasingly prominent among adolescents [1]. The asynchronous development of physical and psychological maturity that occurs during adolescence, coupled with underdeveloped coping mechanisms, contributes to an elevated risk of psychopathology [2]. According to the 2024 Report on Adolescent Mental Health and Academic Status published by the Chinese Academy of Sciences, the screening rates for depression, anxiety, and other emotional disorders have exceeded 25%, indicating a marked trend toward earlier onset, greater severity, and increased complexity. Notably, psychological resilience has been shown to play a critical role in mitigating emotion-related problems under stress/adversity and promoting emotional well-being [3,4].

### Psychological resilience and implicit emotion regulation in adolescents

The conceptualization of psychological resilience in the literature remains inconsistent, encompassing trait-oriented [5,6], outcome-based [7], and process-oriented perspectives [3,8]. These approaches converge on two fundamental components: (a) an exposure to significant adversity and (b) positive adaptation outcomes [9]. Contemporary models therefore conceptualize resilience as a complex, multidimensional construct that incorporates trait, process, outcome, development, and normative dimensions [10].

With respect to the underlying mechanisms of psychological resilience, empirical studies and theoretical models have consistently demonstrated that adolescents with high psychological resilience can better mobilize internal and external resources to buffer against the negative psychological impacts of adversity than those without [11–12]. For example, individuals reporting frequent use of adaptive cognitive emotion regulation strategies exhibit more positive emotional responses and developmental outcomes in the face of stressors [13]. The dual-process theory of psychological resilience further highlights emotion regulation as a critical internal resource for psychological resilience development. This theory posits that psychological resilience emerges through dynamic interactions that occur between explicit/implicit emotion regulation and daily emotional experiences, in which distinct regulatory processes collectively foster adaptive emotional outcomes [14]. Here, explicit emotion regulation refers to the conscious, effortful modulation of emotions as guided by regulatory goals, whereas implicit emotion regulation (IER) involves goal-driven but unconscious/automatic emotional adjustment [15].

The existing studies are predominantly focused on explicit regulatory strategies, with limited attention given to the role of IER in psychological resilience. Compared with explicit regulation, the automaticity of IER confers unique advantages such as unconscious operation, processing efficiency, and real-time responsiveness, which are particularly beneficial under limited cognitive control resources. This is highly relevant to adolescents, whose prefrontal cortical regions that support cognitive control remain underdeveloped [16]. Moreover, IER exerts more profound influences on adolescent psychopathology. Impaired IER functionality has been linked to both anxiety disorders [17] and adolescent depression [18], suggesting its critical role in mental health outcomes.

### The action control theory of emotion regulation and its applications

IER is characterized by automatic processing. However, in goal-directed execution, IER still follows a specific action control sequence. The action control theory of emotion regulation provides a theoretical framework for understanding the effectiveness of IER with respect to psychological resilience. This theory posits that emotion regulation involves complex cognitive processes, in which effective regulation depends on the successful completion of multiple logically interconnected regulatory tasks. Each stage must be adequately fulfilled to ensure successful regulation, and a sequential processing order that primarily includes the goal initiation phase and the strategy implementation phase must be followed [19–20]. The activation of emotion regulation goals can subsequently trigger IER processes [21].

The goal phase of emotion regulation primarily involves the determination of whether and how to regulate emotions. IER goals refer to the orientations of unconsciously pursued emotional state, such as approaching positive emotions or avoiding negative emotions [22]. Although they lack explicit subjective intent, these goals automatically guide emotion regulation behaviors through implicit attitudes. Previous research has examined such goals through the assessment of implicit attitudes toward emotion regulation [19], which stem from individuals’ implicit goals and internalized social norms [23]. These attitudes represent unconscious evaluative tendencies that significantly influence cognition, behavior, and emotional responses during regulation, despite their being inaccessible to introspection [24], profoundly shaping goal formation and execution [23] and serving as a key research focus [25]. The emotion regulation implicit association test (ER-IAT) has been widely used to measure these attitudes [26], and two types have been identified: control-oriented attitudes, which reflect tendencies to regulate negative emotions with positive implicit evaluations, align with “reduce negative emotion” goals and facilitate adaptive strategy selection with reduced cognitive load; and expression-oriented attitudes, which reveal preferences for emotional expression with negative implicit evaluations, correspond to “emotional release” goals and increase the likelihood of direct emotional venting [25–26]. Empirical studies using the ER-IAT have revealed that agreeable university students exhibit control-oriented attitudes [19], whereas shy students display expression-oriented attitudes [27].

The implementation phase of emotion regulation primarily involves the selection of appropriate regulatory strategies and the flexible matching of those strategies to maximize their effectiveness. From the perspective of IER processes, this phase is focused on exploring their protective mechanisms and examining their IER capacities. The core protective mechanism of IER may follow the principle of the automatic counterregulation of attention: when individuals experience a particular emotion, their attention allocation automatically shifts, diverting part of their cognitive resources toward stimuli that carries an opposite emotional valence to that of their current experience, thereby achieving IER [22,28,29]. Experimental procedures for IER typically consist of two stages: the introduction of specific emotions and the triggering of automatic counterregulation [16]. The first stage involves inducing specific emotional states in participants through semantic processing paradigms such as textual reading materials or sentence arrangement tasks [30]. The second stage employs tasks that are designed to elicit automatic counterregulation, which use primarily experimental paradigms, including facial recognition search tasks [19,31] and emotional Stroop tasks [32]. For example, Sun et al. [19] first induced negative emotions in university students through textual reading materials and then administered a face recognition search task to examine the advantageous effect of highly agreeable participants in their IER processes.

### The current study

In summary, this study adopts an integrative perspective on psychological resilience and employs a comprehensive assessment strategy that captures both trait-like psychological resilience factors and adaptive outcome measures. On the basis of the action control theory of emotion regulation, this research presents an examination of the implicit advantages of individuals with high psychological resilience during two distinct phases of implicit emotion regulation: goal initiation and strategy implementation. This approach is aimed at clarifying the significant role of IER in adolescent psychological resilience when coping with daily stressors. Specifically, in Experiment 1, we hypothesized that, compared to adolescents with low psychological resilience, those with high psychological resilience would demonstrate stronger control-oriented implicit attitudes during the goal phase of implicit emotion regulation (H1). In Experiment 2, we hypothesized that after the induction of negative emotions, highly resilient adolescents would have shorter response times when searching for happy faces against a background of angry faces(H2).

## Experiment 1: Advantages of IER attitudes among adolescents with high psychological resilience

Guided by the action control theory of emotion regulation, Experiment 1 was aimed at examining the differences in IER attitudes between adolescents with high and those with low psychological resilience, with the specific objective of identifying any implicit advantages in the goal initiation stage among those with high psychological resilience. Experiment 1 was designed to test H1.

## Materials and methods

### Participants

The various levels of psychological resilience can be differentiated by combining person-centered potential profiling, variable-centered convergent operations and group validity tests, which can be combined to screen for greater validity through mutual testing (see S1- S2 Files). A total of 62 subjects voluntarily participated in behavioral experiment 1. Six subjects were excluded due to an error rate in excess of 20%, resulting in 56 valid subjects (28 in each of the high and low groups). The sample size of 62 was determined to have sufficient statistical power for testing the study hypotheses (see S3 File). The participants were adolescents (age range: 12–15 years; M = 13.05, SD = 0.71; 41.1% female) recruited from two local middle schools between September 8 and November 23, 2022. Written informed consent was provided by all participants’ parents.. This study was approved by the Ethics Committee of Qiqihar Medical University (Approval No. [2021] 123).

### Stimuli

#### The emotion regulation implicit association test (ER-IAT).

The ER-IAT was adapted from the original IAT paradigm [33]. Previous validation studies have demonstrated that the ER-IAT has good psychometric properties [19,26]. The experimental material consisted of four word types [24]. The positive attribute words included excellent, honorable, healthy, beautiful, and comfortable, whereas the negative attribute words included cruel, disgusting, horrible, miserable, and shameful. The emotional control target words included calm, rational, tolerance, restraint, and patience. The emotional expression target words included exuberance, passion, release, reveal, and catharsis.

### Procedure

**Fig 1:**
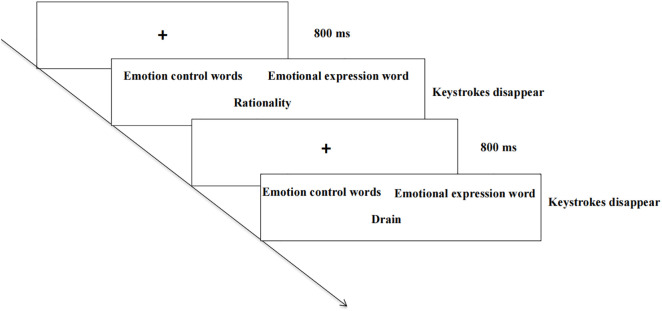
Procedure for the implicit association test of emotion regulation.

The ER-IAT was initiated via a standard seven-step procedure [19,34] with Blocks 1, 2, 3, 5, and 6 serving as practice trials, and Blocks 4 and 7 consisting of the compatible and incompatible test conditions, respectively. The response times and accuracy were recorded for each trial using E-Prime software. The procedure displayed category labels in the upper-left and upper-right screen positions while centrally presenting the stimulus words, with participant response times and accuracy being recorded. The compatible task (Block 4) required classifying “emotional control” and “positive attributes” together (F key) versus a combination of “emotional expression” and “negative attributes” (J key). Conversely, the incompatible task (Block 7) involved pairing “emotional expression” with “positive attributes” (F key) and “emotional control” with “negative attributes” (J key). To control for order effects, the task sequence was counterbalanced across all participants: half of them started with the compatible condition (Blocks 4/7), whereas the other half began with the incompatible condition (Blocks 7/4). The specific experimental workflow is shown in Fig 1.

### Statistical analysis

SPSS 22.0 software was used for data management and analysis. The data were preprocessed, and data with a response time of less than 300 ms or more than 10,000 ms were excluded, as was data with a response error rate of more than 20%. ANOVA was conducted to determine whether a significant interaction effect occurred in the relationship between the psychological resilience level (high/low) and task type (compatible/incompatible) on reaction time.

A D value serves as an index of the participants’ implicit attitudes [34]. A positive D value reflects a positive implicit attitude in emotion regulation and a preference for the deliberate, appropriate control of emotions, whereas a negative D value indicates a negative implicit attitude toward emotion regulation and a preference for the direct expression of emotions [19]. See the S4 File.

## Results

### Differences in implicit attitudes within emotion regulation

**Fig 2:**
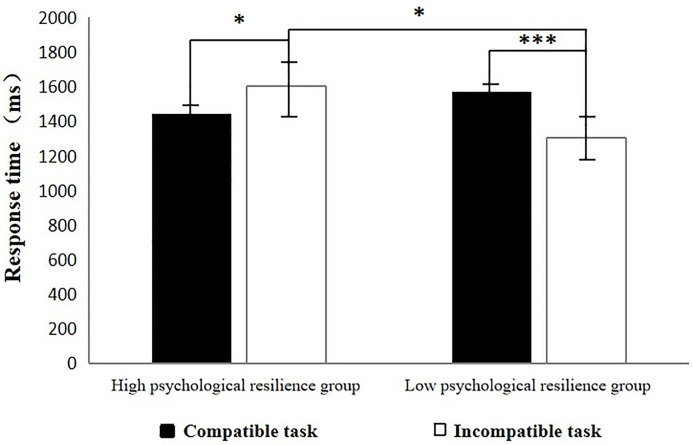
Response times of different psychological resilience groups on compatible and incompatible tasks.

A 2 (experimental task: compatible, incompatible) × 2 (group: high, low psychological resilience group) repeated-measures ANOVA was conducted on response times and revealed a nonsignificant main effect of the experimental task, *F* (1, 54) = 1.18, **p* *= 0.28, and a nonsignificant main effect of the group, *F* (1, 54) = 0.63, *p* = 0.43. However, the interaction effect between the experimental task and the group was significant, *F* (1, 54) = 19.76, *p* < 0.001, *η*_*p*_^2^ = 0.27. Simple effects analyses revealed that the reaction time of the subjects in the high psychological resilience group for the compatibility task was significantly shorter than was their reaction time for the incompatibility task (*F* (1, 54) = 5.64, **p* *= 0.02, *η*_*p*_^2 ^= 0.10). In contrast, the reaction time of the subjects in the low psychological resilience group for the compatibility task was significantly longer than was their reaction time in the incompatibility task (*F* (1, 54) = 15.30, **p* *< 0.001, *η*_*p*_^2^ = 0.22). There was a tendency among the high psychological resilience group for shorter reaction times than those in the low psychological resilience group for the compatibility task, but this difference was nonsignificant, *F* (1, 54) = 1.32, **p* *= 0.26. In the incompatibility task, the high psychological resilience group exhibited significantly longer reaction times than the low psychological resilience group did, *F* (1, 54) = 5.66, **p* *= 0.02, *η*_*p*_^2^ = 0.10. The results of the descriptive statistics are shown in Table 1 and Fig 2.

**Table 1:** Reaction times of groups with different psychological resilience on two types of tasks (*M*±*SD*) (ms).

Experimental tasks	High psychological resilience	Low psychological resilience
**Compatible task**	1440.66 ± 421.36	1567.59 ± 404.67
**Incompatible task**	1601.57 ± 467.33	1302.43 ± 473.90

An independent-samples *t* test was subsequently conducted on the D values of the groups with high and those with low psychological resilience, and it revealed a significant difference between these two groups, *t* (54) = 4.45, **p* *= 0.001, *Cohen’s d* = 1.17, *95% CI* = [0.20, 0.52]. The D values of the subjects in the high psychological resilience group (0.13 ± 0.30) were significantly greater than those of the subjects in the low psychological resilience group (−0.22 ± 0.30).

## Discussion

The experimental results reveal that those participants with high psychological resilience had significantly shorter reaction times in the compatible task than they did in the incompatible task, which is consistent with the IAT hypothesis. Moreover, their reaction times in the compatible task were also shorter than those of the participants with low psychological resilience. This finding indicates that the participants with high psychological resilience more readily associated emotional control with positive attributes and emotional expression with negative attributes; in other words, they implicitly viewed emotional control as positive and emotional expression as negative. As a control for the potential influence of cognitive processing speed on the IAT effect [34], further analysis was conducted and revealed that D scores were significantly greater than zero for those participants with high psychological resilience but less than zero for those participants with low psychological resilience, with a significant between-group difference that supports Hypothesis 1. These findings suggest that the participants with high psychological resilience tended to hold an implicit control-oriented emotion regulation attitude, whereas the participants with low psychological resilience exhibited an expression-oriented implicit regulation attitude. Those individuals with positive implicit attitudes toward emotion regulation automatically adjusted their emotions according to situational demands in negative contexts, whereas those with negative implicit attitudes showed unmodulated emotional expression without automatic regulation [24].

## Experiment 2: Enhanced implicit regulation efficacy in adolescents with high psychological resilience

Experiment 2 extended these findings through an investigation of the group differences in IER abilities, with a focus on the presence of implicit advantages during the process implementation stage for adolescents with high psychological resilience.To follow up on the findings of Experiment 1 and to test H2, we conducted Experiment 2.

## Materials and methods

### Participants

A total of 76 subjects were selected to voluntarily participate in behavioral experiment 2. Participants were recruited from two local middle schools between September 8 and November 23, 2022. One subject was excluded because their error rate exceeded 20%, resulting in 75 valid subjects (high group 38, low group 37). The sample size of 75 was determined to have enough statistical power for testing the study hypotheses (see S3 File).They were aged 12–15 years (M = 13.25, SD = 0.88), and 50.7% were female.

The grouping criteria for the high/low psychological resilience groups, the participant age ranges and the task compensation methods in Experiment 2 were identical to those employed in Experiment 1. This study was approved by the Ethics Committee of Qiqihar Medical University (Approval No. [2021] 123), and written informed consent was obtained from all participants’ parents.

### Stimuli

#### Negative emotion-inducing experimental materials.

A 3-minute clip of the movie “Better Days,” which was about a heroine who was bullied in school, was used to induce negative emotions.

The effects of negative stimulus elicitation on subjects’ emotional experience (see S5 File).

#### Facial expression images in the visual search task.

Six facial expression images were selected from the Chinese Affective Picture System (CAPS) [35], including two happy, one angry, and one neutral facial images as well as one male facial image and one female facial image. A 3 × 3 matrix containing nine facial images was designed [19]. Half of the matrices contained eight identical distractor images of facial expressions and one different target facial expression. For example, the happy-angry category had 1 happy image and 8 angry images. The other half of the matrices contained nine identical facial expression images. The negative emotion-induced visual search task used happy-neutral, angry-neutral, and happy-angry target-interference facial expression combination matrices. The facial expression matrices for men and those for women were designed to control for the effect of sex on the experiment. Each subject completed 108 trials.

### Procedure

The procedure comprised five sequential phases: (a) a baseline state measure of emotion was taken; (b) the emotional potency and intensity of the subjects were evaluated at this point; (c) the subjects watched a movie clip of induced negative emotion; (d) the post-induced emotional potency and intensity of the subjects were evaluated; and (e) the subjects performed a visual search task for facial expressions.

To balance the influence of the order effect on the experimental results, half of the subjects were told to press “f” if the faces were judged to be identical and “j” if they were not, whereas the other half of the subjects were asked to press the opposite keys.

### Statistical analysis

All the data were processed and analyzed using SPSS 22.0. First, paired-samples t tests were used to compare the pleasure and arousal ratings between the baseline and postelicitation phases within each resilient group (higher vs. lower). We then conducted a 2 (Group: high psychological resilience, low psychological resilience) × 3 (Facial Expression Combination: happy-angry, happy-neutral, angry-neutral) mixed-design ANOVA to examine the main and interaction effects, as well as conducting follow-up simple effects analyses where appropriate.

## Results

A 2 (group: high and low psychological resilience) × 3 (face combination mode: happy-angry, happy-neutral, angry-neutral) repeated-measures ANOVA was conducted. A significant main effect of the grouping was found, *F* (1, 73) = 7.54, *p* < 0.01, *η*_*p*_² = 0.10, with the high psychological resilience group having a shorter reaction time than the low psychological resilience group. There was also a significant main effect of the facial combination mode, *F* (2, 146) = 13.51, *p* < 0.001, *η*_*p*_² = 0.16, with the angry-neutral reaction time being significantly longer than either the happy-neutral or the happy-angry reaction times were (*p* < 0.001), and the difference between the happy-neutral and happy-angry reaction times was nonsignificant (*p* > 0.05). The interaction effect between the facial combination mode and group was nonsignificant, *F* (2, 146) = 0.55, **p* *= 0.56. Further independent-sample t tests revealed a significant difference between the reaction times of the two groups for the happy-angry face combination, *t* (73) = −2.86, **p* *< 0.01, *Cohen’s d* = −0.66*, 95% CI* = [−367.40, −65.91]. The subjects with high psychological resilience levels had a significantly shor*t*er reaction time when searching for happy faces than did the subjects with low psychological resilience levels. In addition, subjects with high levels of psychological resilience had significantly shorter response times than did subjects with low levels of psychological resilience when searching for happy and angry faces among neutral faces, *t* (73) = −3.02, **p* *< 0.01, *Cohen’s d* = −0.69, *95% CI* = [−346.54, −71.12]; *t* (73) = −2.21, **p* *= 0.03, *Cohen’s d* = −0.51, *95% CI* = [−350.78, −17.91]. The descriptive statis*t*ics for the reac*t*ion times to the three types of face combinations are shown in Table 2.

**Table 2:** Comparison of response time for facial expressions evoked by negative emotions (M±SD) (ms).

Face combination	High psychological resilience	Low psychological resilience
**Happy-Angry**	1363.73 ± 229.82	1580.38 ± 383.41
**Happy-Neutral**	1356.11 ± 201.96	1565.12 ± 356.16
**Angry-Neutral**	1450.10 ± 287.96	1630.02 ± 408.09

## Discussion

Experiment 2 employed an IER paradigm that combined emotional induction procedures and facial visual search tasks to examine the regulatory effects on participants with different psychological resilience. The measurement results confirmed the successful induction of negative emotional experiences, as manifested by significantly lower postinduction pleasure ratings and higher arousal levels than those at baseline in both groups, which aligns with previous findings regarding the efficacy of film clips in emotion elicitation [36].

Notably, participants with high psychological resilience demonstrated significantly shorter reaction times when happy faces were detected among angry faces than did their counterparts with low psychological resilience. As proposed in prior research [37], an enhanced implicit positive affect can improve the detection of happy faces among angry face arrays, suggesting that increased implicit positivity during emotional recovery is related to implicit regulation processes. Thus, our results indicate that participants with high psychological resilience likely benefit from this implicit positive affect restoration mechanism.

## General discussion

Building on the action control theory of emotion regulation, which posits that effective emotion regulation involves the execution of sequential regulatory processes, two experiments were conducted in this study to examine the implicit advantages of emotion regulation among adolescents with high levels of psychological resilience. Experiment 1, in which the ER-IAT test was employed, revealed that adolescents with high psychological resilience exhibited a control-oriented implicit attitude toward emotion regulation, whereas their counterparts with low psychological resilience displayed an expression-oriented implicit attitude. In Experiment 2, following negative emotion induction, the participants completed a face-search task, in which adolescents with high psychological resilience detected happy faces among angry faces significantly faster than did those with low psychological resilience. These findings collectively indicate that executive control in IER is closely associated with psychological resilience. Under negative emotional states, the positive implicit attitudes of adolescents with high psychological resilience may automatically guide the goal orientation of implicit regulation through the dynamic mechanism of automatic reverse regulation, thereby enhancing the implicit positive affect. These findings suggest that IER plays a critical role in fostering the development of psychological resilience at the behavioral level.

### Control-oriented IER attitudes facilitate psychological resilience in adolescents

Experiment 1 revealed that adolescents with high levels of psychological resilience tend to hold control-oriented implicit attitudes toward emotion regulation, preferring moderate emotional control over unrestrained expression. Research has consistently demonstrated the significance of control-oriented implicit regulation attitudes in mitigating a negative affect and promoting emotional well-being. Compared with those holding expression-oriented implicit attitudes, individuals with control-oriented implicit attitudes exhibit more adaptive responses across emotional experiences and physiological arousal in the face of negative situations [25,26], which suggests that automatic regulation processes result in attenuated emotional responses [24,38]. Furthermore, Hopp et al.‘s [39] study, which used the Emotion Regulation Implicit Association Test, confirmed the associations among control-oriented implicit attitudes, self-reported health, and the conscious use of cognitive reappraisal strategies. These findings collectively indicate that control-oriented implicit attitudes may play a crucial role in the development of psychological resilience. However, the adaptive value of such attitudes can vary significantly across age, sex, and sociocultural context [24].

### IER capacity facilitates psychological resilience in adolescents

Experiment 2 demonstrated that following negative emotion induction, those participants with high psychological resilience detected happy faces among angry face arrays significantly faster than did those with low psychological resilience. Previous research has indicated that reaction times for identifying happy faces among angry (or neutral) faces reflect emotion regulation capacity, as successful detection requires the disengaging of attention from the emotional background and the shifting of focus to the target face—with faster disengagement indicating a stronger regulatory ability [40]. Thus, these findings suggest that adolescents with high psychological resilience may more effectively overcome the “anger superiority effect”, disengage their attention from dominant negative stimuli, enhance their implicit positive affect, and facilitate automatic emotional recovery following negative emotion induction.

The enhanced implicit recovery observed in adolescents with high psychological resilience may be explained by their adherence to the automatic reverse regulation principle during the process of IER [29]. This cognitive mechanism enables such adolescents to transcend their currently dominant emotional state and automatically allocate attentional resources to stimuli with an opposing valence. This flexible regulatory pattern essentially serves as an efficient self-protective mechanism [16] and offers greater adaptive value for emotional well-being than general positivity biases do [41]. Additionally, the inherent advantage of individuals with high psychological resilience in maintaining motivational states for implicit regulation likely contributes to this effect. Collectively, these findings suggest that adolescents with high psychological resilience exhibit automated and flexible implicit regulatory capacities that play a pivotal role in the fostering of psychological resilience during daily stressors.

Collectively, the findings from both experiments consistently demonstrate a close association between executive control in IER and psychological resilience. In line with previous research, implicit emotion regulation—as an unconscious cognitive-affective process—may constitute a fundamental component of self-regulation and mental health [42]. As proposed by Prout et al. [43] in their regulation-focused psychotherapy for children (RFP-C) model, improving the IER system in children with low psychological resilience can foster adaptive functioning. This underscores that emotion regulation under adversity or daily stress serves as a critical resource for fostering psychological resilience: adolescents with high psychological resilience not only employ adaptive explicit strategies when coping with challenges [1] but also have advantages in implicit regulation. These automatic and effortful regulatory capacities likely jointly contribute to an enhanced positive affect and emotional affect [44–45].

IER has garnered increasing attention, with the evidence suggesting that it may represent the most direct scientific bridge to psychological resilience research [43]. In mental health promotion, implicit mechanisms might surpass explicit regulation in importance [15]. However, Studies examining the linkages between psychological resilience and implicit regulation remain scarce. Grounded in action control theory, this study systematically elucidates the proactive role of IER in psychological resilience development. The findings suggest that IER ability may serve as a critical internal resource that promotes psychological resilience in adolescents, highlighting its significance for maintaining emotional health under daily stress. First, applications in educational and preventive settings. IER training (e.g., reinforcing associations between emotion control words and positive words) can be integrated into school-based mental health education programs. Combined with group interventions such as mindfulness meditation and cognitive restructuring, such training could simultaneously improve both conscious and unconscious emotion regulation patterns, thereby enhancing adolescents’ adaptability to daily adversity. Additionally, this study facilitates the identification of high-risk adolescents for early intervention. Individuals with low psychological resilience and weak implicit regulation abilities can be screened in schools, or hospitals settings using cognitive bias modification (CBM) or implicit association training paradigms. These approaches help reshape implicit emotional processing pathways and enhance automatic processing of positive emotional stimuli. This study demonstrates potential value in advancing precise prevention and intervention strategies for promoting emotional health in adolescents.

The present study has several limitations. First, the film clips lacked standardized normative ratings and administration protocols, which potentially limits their cross-study comparability. Second, conducting t tests for each facial combination despite nonsignificant ANOVA interactions risks Type I error inflation—a limitation that requires future verification. Third, neurophysiological investigations are needed to explicate the protective neural mechanisms of implicit regulation for psychological resilience.

## Conclusions

Building on the action control theory of emotion regulation, adolescents with high psychological resilience may possess implicit advantages in both the goal setting and implementation phases of emotion regulation. These findings underscore the role of IER as a critical resource in the fostering of psychological resilience during adolescence.

## Supporting information

S1 File Approach to psychological resilience grouping. (PDF)

S2 File Scales involved in the study. (PDF)

S3 File Calculation of minimum sample size. (PDF)

S4 File Calculation of the D-value, an indicator of IER attitudes. (PDF)

S5 File Effects of negative stimulus elicitation on subjects’ emotional experience.
(PDF)

S6 Data (XLSX)
