# Supplementary material for: Implicit benefits of adolescents with high psychological resilience in action control of emotion regulation
Source: PLoS One. 2025 Sep 16;20(9):e0332384. doi: 10.1371/journal.pone.0332384 (PMC12440164; doi:10.1371/journal.pone.0332384)
Supplement: S1 File — (PDF) [file pone.0332384.s001.pdf]

S1. Approach to psychological resilience grouping

After potential profile analysis, 534 adolescents were potentially profiled based on the Psychological Resilience Scale question items, and 1-5 potential categories were extracted. The results for each indicator are shown in Table S1. The entropy indicator was 0.80 when the number of categories was 2, indicating that the representative classification accuracy was more than 90% (Hu et al., 2017). The results of the LMRT also showed that the 2-category model was better than the 1-category model, $p<0.001$, and the improvement in optimization obtained by indicators such as the AIC, BIC, and ssaBIC varied less with increasing category number in the model after category number 2. Although the difference between the category 3 and 4 models is significant at the 0.05 level, their entropy metrics begin to decline. Therefore, the 2-category model was deemed to describe the type information more adequately; the final numbers of high and low groups were determined to be 275 and 268, respectively; and the attribution probability matrices of the two potential categories were 0.93 and 0.96, respectively.

The convergence operation method First, the total stress scores of all the adolescents were ranked in order of high to low stress. According to the criteria of previous research (Lv Mengshi et al., 2017), the top 50% of the participants were considered "potentially psychologically resilient". A total of 267 participants (128 males and 139 females) fulfilled the preconditions for the definition of psychological resilience. The participants were further ranked according to their subjective well-being from high to low scores, in which the initial selection criteria for the high psychological resilience group were 27% of the top scores in terms of subjective well-being, totaling 60 participants, and the low psychological resilience group was 27% of the bottom scores in terms of subjective well-being, totaling 56 participants.

Group validity test Comprehensive convergence of the results of the operational method and potential profile analysis were tested. Among the 54 participants in the high psychological resilience group and 50 participants in the low psychological resilience group, The validity of screening the high and low groups was then verified using the Adolescent Psychological Resilience Scale, $t = 26.25$, $p < 0.001$.

Calculation of minimum sample size Sample size was determined a priori by running a priori power analysis using G*Power 3.1, a medium effect size of 0.5 in repeated measures ANOVA design with a power ($1-\beta$) set at .80 and αset at .05. Experiment 1 used a 2*2 mixed experimental design that required a minimum of 17 subjects per group, and Experiment 2 used a 2*3 mixed experimental design that required a minimum of 21 subjects per group. To ensure the robustness of the experimental results, while maintaining consistency with comparable IAT studies (e.g., Sun et al., 2019), Experiment 1 ultimately included 62 participants, Experiment 2 ultimately included 75 participants.
