# Supplementary material for: Implicit benefits of adolescents with high psychological resilience in action control of emotion regulation
Source: PLoS One. 2025 Sep 16;20(9):e0332384. doi: 10.1371/journal.pone.0332384 (PMC12440164; doi:10.1371/journal.pone.0332384)
Supplement: S2 File — (PDF) [file pone.0332384.s002.pdf]

S2 Scales involved in the study

2.1 Adolescent life events scale

The scale was developed for secondary school students and asks participants to indicate whether the events on the scale had occurred and about their degree of influence in the past 12months; this scale includes a total of five factors and 26 items (Xin and Yao, 2015). The internal consistency reliability of the scale was 0.91.

2.2 Trait psychological resilience scale

The Connor-Davidson-10 by Campbell-Sills and Stein (2007) was used; it has 10 items with high reliability and validity. The internal consistency reliability of the scale was 0.83.

2.3 Adolescent psychological resilience scale

The scale measures psychological resilience in adolescents and was developed based on traditional Chinese culture. The scale consists of 27 items and contains two factors (Hu and Gan, 2008).

2.4 Subjective happiness index scale

This scale contains nine items, eight of which constitute the overall affective index, one of which is the life satisfaction index, and the weighted sum of the two is the overall happiness index (Wang et al., 1999). The internal consistency reliability of the scale was 0.872.
