# Supplementary material for: Implicit benefits of adolescents with high psychological resilience in action control of emotion regulation
Source: PLoS One. 2025 Sep 16;20(9):e0332384. doi: 10.1371/journal.pone.0332384 (PMC12440164; doi:10.1371/journal.pone.0332384)
Supplement: S3 File — (PDF) [file pone.0332384.s003.pdf]

S3. Calculation of minimum sample size

Sample size was determined a priori by running a priori power analysis using G*Power 3.1, a medium effect size of 0.5 in repeated measures ANOVA design with a power (1-β) set at .80 and αset at .05. Experiment 1 used a 2*2 mixed experimental design that required a minimum of 17 subjects per group, and Experiment 2 used a 2*3 mixed experimental design that required a minimum of 21 subjects per group. To ensure the robustness of the experimental results, while maintaining consistency with comparable IAT studies (e.g., Sun et al., 2019), Experiment 1 ultimately included 62 participants, Experiment 2 ultimately included 75 participants.
