# Supplementary material for: Implicit benefits of adolescents with high psychological resilience in action control of emotion regulation
Source: PLoS One. 2025 Sep 16;20(9):e0332384. doi: 10.1371/journal.pone.0332384 (PMC12440164; doi:10.1371/journal.pone.0332384)
Supplement: S4 File — (PDF) [file pone.0332384.s004.pdf]

S3 Calculation of the D-value, an indicator of implicit emotion regulation attitudes

Greenwald et al. (2003) proposed that D-value as an indicator of subjects' implicit attitudes, The D-value is calculated by dividing the difference between the response time on the compatible task and the response time on the incompatible task by the standard deviation of the correct response time on both parts.

In addition, D values differed significantly between grade levels, $t\ (54) = 3.58$，$p < 0.01$， *Cohen's* $d = 1.05$， $95\%CI = [0.15, 0.53]$, The D-value of the first year (0.05±0.30) was significantly greater than that of the second year (−0.29 ± 0.34).
