# Supplementary material for: Implicit benefits of adolescents with high psychological resilience in action control of emotion regulation
Source: PLoS One. 2025 Sep 16;20(9):e0332384. doi: 10.1371/journal.pone.0332384 (PMC12440164; doi:10.1371/journal.pone.0332384)
Supplement: S5 File — experience. (PDF) [file pone.0332384.s005.pdf]

S4. Effects of negative stimulus elicitation on subjects' emotional experience

In order to examine whether the experimental materials successfully evoked negative emotional experiences in the subjects, paired-samples t-tests were conducted on the degree of pleasure (rated on a scale of 1-7) and the degree of arousal (rated on a scale of 1-7) of the emotional experiences of the subjects in both groups at baseline and post-evocation. Results revealed that baseline scores were significantly higher than post-evoked scores in the high psychological resilience group on the pleasure scale, $t\ (37) = 11.58$, $p < 0.001$, *Cohen's d* = 2.65, 95%*CI* = [2.37, 3.37]， and that baseline scores were significantly higher than post-evoked scores in the low psychological resilience group, $t\ (36) = 8.02$， $p < 0.001$, *Cohen's d* = 1.65, 95%*CI* = [1.25, 2.10]; the high psychological resilience group also had significantly higher post-evoked scores than baseline scores on the Emotional Experience Feeling Intensity scale, $t\ (37) = 6.77$, $p < 0.001$, *Cohen's d* = 1.09, 95%*CI* = [1.20, 2.22], and the low psychological resilience group had significantly higher post-evoked scores than baseline scores as well, $t\ (36) = 3.72$, $p < 0.01$, *Cohen's d* = 0.81, 95%*CI* = [0.49, 1.67]. In addition, independent samples t-tests were performed on the pleasure and arousal scores of the emotional experiences of the two groups of subjects at baseline and post-evocation. The results revealed that the high psychological resilience group had higher pleasure than the low psychological resilience group at the baseline time point $t\ (73) = 4.58$, $p < 0.001$, *Cohen's d* = 1.06, 95%*CI* = [0.62, 1.57], but there was no significant difference in arousal ($p > 0.05$); there was no significant difference between the two groups of subjects in terms of pleasure and arousal scores following the induction of negative emotional stimuli （$p > 0.05$).
